# Immunological profiling of rheumatoid factor-positive primary Sjögren’s syndrome by single-cell RNA sequencing

**DOI:** 10.3389/fimmu.2026.1822615

**Published:** 2026-06-11

**Authors:** Yifei Wang, Wenqi Ji, Fei Teng, Yuxin Han, Heng Xu, Yuxing Zhi, Qingwen Tao, Guangyao Chen

**Affiliations:** 1 Dongzhimen Hospital, Beijing University of Chinese Medicine, Beijing, China; 2 Graduate School, Beijing University of Chinese Medicine, Beijing, China; 3 Qihuang School, Beijing University of Chinese Medicine, Beijing, China; 4 School of Life Sciences, Beijing University of Chinese Medicine, Beijing, China; 5 Department of Traditional Chinese Medicine (TCM) Rheumatology, China-Japan Friendship Hospital, Beijing, China

**Keywords:** cell communication, primary Sjögren’s syndrome, pseudo-time analysis, rheumatoid factor, single-cell RNA sequencing

## Abstract

**Objective:**

Primary Sjögren’s syndrome (pSS) is a highly heterogeneous autoimmune disease. Previous studies have suggested that rheumatoid factor (RF)-positive patients appear to have a higher incidence of systemic involvement. However, the underlying mechanisms remain unclear. This study aims to elucidate the potential biological mechanisms through single-cell RNA sequencing (scRNA-seq).

**Methods:**

Peripheral blood mononuclear cells from 3 healthy controls, 6 RF-negative (RF^-^) pSS patients, and 6 RF-positive (RF^+^) pSS patients were subjected to scRNA-seq. Subsequent analyses included pathway enrichment analysis, trajectory analysis, cell-cell communication analysis, and B cell receptor (BCR) and T cell receptor (TCR) repertoire profiling. Potential differences were further validated by flow cytometry.

**Results:**

Analysis of B cell subsets revealed a significantly increased proportion of plasma cells in RF^+^ patients, and differential gene expression analysis indicated enhanced pathways related to antibody production, clonal expansion, and type I interferon activation. In T-cell subsets, the RF^+^ group showed a notably higher proportion of CD8^+^ T cells, which exhibited stronger cytotoxicity and activation of interferon signaling. In contrast, mucosal-associated invariant T (MAIT) cells demonstrated activated interferon signaling but were present at a lower proportion compared to the RF^-^ group. Cell–cell communication analysis revealed strengthened crosstalk between B cells and T cells in the RF^+^ group. Pseudo-time trajectory analysis further indicated that in RF^+^ patients, both plasma cells and CD8^+^ T cells exhibited a developmental skew toward a terminally differentiated state. Additionally, both BCR and TCR repertoire analyses indicated RF^+^ and RF^-^ patients exhibiting distinct patterns in clonal distribution and frequency. Flow cytometry further validated the increased proportion of CD8^+^ T cells and the decreased proportion of MAIT cells in the peripheral blood of RF^+^ pSS patients.

**Conclusion:**

The RF status in pSS patients is closely associated with specific immune cell profiles. RF^+^ patients exhibit more active plasma cell responses, enhanced CD8^+^ T-cell cytotoxicity and interferon responses, as well as heightened interferon signaling yet reduced proportion of MAIT cells. RF status serves as a key stratification marker for immune heterogeneity in pSS patients, providing new insights for understanding disease mechanisms and developing targeted therapeutic strategies.

## 1 Introduction

Primary Sjögren’s syndrome (pSS) is a chronic, systemic autoimmune disorder characterized by lymphocytic infiltration of exocrine glands, leading to the hallmark symptoms of dry eyes and dry mouth (1, 2). Beyond this sicca syndrome, pSS manifests with considerable heterogeneity, encompassing a broad spectrum of extraglandular systemic complications and a markedly increased risk of B-cell lymphoma (3, 4). This clinical and pathophysiological diversity suggests the existence of distinct molecular endotypes within the pSS patient population. Among the classic serological markers, rheumatoid factor (RF) positivity is frequently observed in pSS and has been associated with more severe disease phenotypes, including higher systemic disease activity, greater focus scores on salivary gland biopsies, and an increased prevalence of cryoglobulinemia and lymphoma (5–11). However, the precise cellular and molecular mechanisms that underlie this RF-positive subgroup and drive its more aggressive clinical course remain poorly understood, limiting the development of targeted therapeutic strategies.

Recent advancements in single-cell RNA sequencing (scRNA-seq) technology offer an opportunity to deconstruct this heterogeneity at a higher resolution (12). By enabling the simultaneous analysis of gene expression profiles across thousands of individual cells within complex tissues, scRNA-seq can identify novel cellular subsets, delineate abnormal cell states, and reconstruct pathogenic interaction networks within the immune microenvironment. Current research comparing RF-positive (RF^+^) and RF-negative (RF^-^) pSS patients remains largely limited to descriptive associations. Applying scRNA-seq to the study of pSS holds the potential to move beyond this limitation. This approach allows for the direct examination of specific immune cell populations that differ quantitatively or qualitatively in RF-positive patients (13, 14). Therefore, this study aims to utilize scRNA-seq to characterize and compare the peripheral blood immune landscapes of RF-positive versus RF-negative pSS patients. Through this analysis, we seek to identify defining cellular features, key driver pathways, and potential therapeutic targets specific to this clinically relevant subgroup.

## 2 Materials and methods

### 2.1 Study subjects

For the scRNA-seq component, 6 RF^+^ and 6 RF^-^ pSS patients, together with 3 healthy medical staff as controls (HC), were enrolled from China-Japan Friendship Hospital. Additionally, a separate cohort of 6 RF^+^ and 6 RF^-^ patients was recruited for validation by flow cytometry and quantitative real-time polymerase chain reaction (qPCR). The diagnosis of pSS was based on the 2016 classification criteria established by the American College of Rheumatology (ACR) and European League Against Rheumatism (EULAR) (15). RF was measured using an immunoturbidimetric assay by the Department of Laboratory Medicine of China-Japan Friendship Hospital. A level of ≥ 20 IU/mL was defined as positive, while a level of < 20 IU/mL was defined as negative. Exclusion criteria comprised individuals with a history of medication use within 3 months and other systemic autoimmune diseases (such as systemic lupus erythematosus, dermatomyositis, rheumatoid arthritis, adult-onset Still’s disease), severe cardiovascular disease, infectious diseases, hematologic conditions, malignancies, neuropsychiatric illnesses, pregnancy, or other significant comorbidities. The study was approved by the Ethics Committee of China-Japan Friendship Hospital (Approval KY2025-294-01), and written informed consent was obtained from all participants.

### 2.2 Materials and antibodies

Ficoll-Paque Premium (17544602, GE Healthcare, USA), Red Blood Cell Lysis Solution (130-094-183, Miltenyi Biotech, GER), Chromium Single Cell Human B cell receptors (BCR) Amplification Kit (PN:1000253, 10x Genomics, USA), Chromium Single Cell Human T cell receptors (TCR) Amplification Kit (PN:1000252, 10x Genomics, USA), 10x Chromium Single Cell Kit (v2 PN:1000263, 10x Genomics, USA), MACS^®^ SmartStrainers (70 µm, 130-098-462, Miltenyi Biotech, GER), MACS^®^ SmartStrainers (30 µm, 130-098-458, Miltenyi Biotech, GER), Dead Cell Removal Kit (130-090-101, Miltenyi Biotech, GER), Phosphate buffer saline (PBS)(10010023, Invitrogen, USA), RPMI Medium 1640 (31800, Solarbio, China), PerCP/Cyanine5.5 anti-human TCR Vα7.2 Antibody (351710, Biolegend, USA), BD OptiBuild™ BV421 Mouse Anti-Human CD161 (748286, BD Biosciences, USA), APC anti-human CD3 Antibody (300312, Biolegend, USA), FITC anti-human CD8a Antibody (300906, Biolegend, USA), Reverse Transcription System (A3500, Promega, USA), SYBR Green Realtime PCR Master Mix (QPK-201, TOYOBO, Japan).

Primer sequences for PCR amplification are shown in **Table 1**, which were synthesized by Beijing Qingke Biotechnology Co., Ltd.

**Table 1:** Sequences of PCR primers.

Gene	Primer sequence (5′to3′)
Human *IFI44L*	Sense: TGCACTGAGGCAGATGCTGCG
	Antisense: TCATTGCGGCACACCAGTACAG
Human *GAPDH*	Sense: GGAGCGAGATCCCTCCAAAAT
	Antisense: GGCTGTTGTCATACTTCTCATGG

### 2.3 PBMC isolation and purification

Peripheral blood mononuclear cells (PBMCs) were isolated using density gradient centrifugation with Ficoll-Paque Premium. Venous blood was collected from each participant into BD Vacutainer tubes containing ethylenediaminetetraacetic acid (EDTA) and diluted 1:1 with 1 × PBS. The diluted blood sample was gently layered onto the surface of the Ficoll separation solution, ensuring a clear interface between the two liquids. After centrifugation (500g, room temperature, 30 minutes, with low acceleration and brake settings), the PBMC layer was carefully aspirated. RPMI 1640 medium was added to resuspend the cells, followed by another centrifugation step (4 °C, 400 × g, 5 minutes). The supernatant was discarded, and the cells were resuspended. Red blood cell lysis buffer was added, and the mixture was incubated on ice for 3 minutes. Subsequently, the sample was centrifuged again (400g, 4 °C, 5 minutes), and the supernatant was discarded. This washing step was repeated until the cell pellet appeared free of red color.

### 2.4 Single-cell suspension preparation

Single-cell suspensions were sequentially filtered through 70-μm and 30-μm cell strainers. Cell viability was assessed using Countstar Rigel fluorescence-based cell analyzer. Only preparations with >90% viability were used for subsequent experiments, and dead cells were subsequently removed with a Dead Cell Removal Kit based on the viability results. Finally, cells were resuspended in 1x PBS supplemented with 0.04% bovine serum albumin at a concentration of 700–1200 cells/μL and processed for scRNA-seq following the manufacturer’s protocol at Novogene Bioinformatics Technology Co., Ltd (Tianjin, China).

### 2.5 Single-cell library preparation and sequencing

Single-cell libraries were prepared using the 10× Genomics platform. Cell suspensions were loaded onto a 10x Chromium Chip and processed with a 10x Chromium Controller for cell barcoding according to the manufacturer’s protocol. Following barcoding, cellular RNA was reverse-transcribed, amplified, and converted into sequencing libraries using the 10× Library Construction Kit. For immune receptor profiling, VDJ libraries were constructed from the barcoded cDNA using the species-specific 10× BCR and 10× TCR Amplification Kit. All libraries were sequenced on an Illumina NovaSeq platform with 150-bp paired-end reads.

### 2.6 Sequencing data processing and quality control

Raw FASTQ files generated by 10×Genomics were aligned and quantified using Cell Ranger (v4.3.0). The Read10X function was used to import the gene expression matrices into R environment. Seurat objects were constructed using the Seurat package (v4.4.0). Furthermore, cells meeting the following criteria were retained: the number of detected genes was between 300 and 7,000, the mitochondrial gene percentage was below 20%, the hemoglobin gene percentage was below 5%, and the total UMI count was below 20,000. The DoubletFinder package (v2.0.3) was then applied to identify and remove doublets (16).

### 2.7 Dimensionality reduction, clustering, and cell group annotation

The data passing quality control were first assessed for cell cycle scores (S and G2M) using the CellCycleScoring function. After library-size normalization, the top 2,000 highly variable genes were selected via FindVariableFeatures with the variance-stabilizing transformation (vst) method (17). The data were then scaled, during which the S.Score and G2M.Score were regressed out to remove cell cycle-associated variation. After dimensionality reduction by principal component analysis (PCA), Harmony integration was performed with the harmony package (v1.2.4) on the cleaned dataset to correct for technical batch effects, using the first 20 principal components and sample origin as the batch covariate. A shared nearest neighbor (SNN) graph was subsequently constructed based on the 20 Harmony-corrected dimensions (18). Clustering was performed using the FindNeighbors and FindClusters functions, followed by visualization with UMAP. Clusters were manually annotated based on an integrated assessment of the existing literature, established canonical cell-type markers, and cluster-specific marker genes (19). For each major cell type, further normalization, dimensionality reduction, batch effect correction with Harmony, and clustering were performed. To enable detailed characterization, the immune cell data were stratified into discrete T cell and B cell subsets for independent analysis. Subset identification and annotation relied on specific antibody signatures and functional markers, enabling subsequent calculation of subset-specific proportions. Differentially expressed genes (DEGs) for T cell subsets were identified using the nonparametric Wilcoxon rank-sum test via the FindMarkers function, with screening criteria set as: |log_2_FC| > 0.25, expression of the gene in at least 10% of cells, and an adjusted p-value < 0.05. For B cell subset DEG screening, the RF^+^ group was first downsampled 20 times to balance the cell numbers between RF^+^ and RF^-^ groups. In each iteration, DEGs were identified using the Wilcoxon rank-sum test via FindMarkers (single-iteration criteria: no initial threshold for log_2_FC, gene expressed in at least 5% of cells, detected in at least 3 cells, and expressed in at least 3 cells per group). The final criteria for screening stable DEGs were: average |log_2_FC| > 0.25, consistency in the direction of differential expression ≥80%, and a *P*-value < 0.05 in ≥ 30% of the iterations.

### 2.8 Pathway enrichment analysis

Functional enrichment analysis of significant DEGs was conducted for B and T cell subset. Gene symbols were converted to Entrez IDs using the org.Hs.eg.db database. Gene Ontology (GO) enrichment across biological processes, cellular components, and molecular functions was performed using the enrichGO function from the clusterProfiler (v4.16.0) package, with a significance threshold of p < 0.05. Kyoto Encyclopedia of Genes and Genomes (KEGG) pathway analysis was executed using the enrichKEGG function from the clusterProfiler package, specifying the human organism (20). Enrichment results were visualized using bar plots, dot plots, and circos plots generated with ggplot2 (v4.0.1) and circlize (v0.4.17) packages. All analyses were performed in R.

### 2.9 Cell-cell communication network analysis

Cell-cell communication analysis was performed using the R package CellChat (v2.2.0) (21). Data pre-processing involved the identification of overexpressed genes and interactions, followed by data smoothing utilizing the human protein-protein interaction network. Cell-cell communication probabilities were calculated using the computeCommunProb function, and interactions involving fewer than 3 cells were filtered out. To dissect the specific communication between B cells and T cells, four distinct interaction categories were extracted: intra-B cell communication, intra-T cell communication, directed communication from B cells to T cells, and directed communication from T cells to B cells. All results were saved in CSV format for subsequent quantitative analysis. Overall interaction circle plots were generated to visualize the number and strength of interactions among all cell subpopulations. The CellChatDB.human database was employed, focusing on secreted signaling pathways.

### 2.10 Single‐cell trajectory analysis

T-cell and B-cell subsets were extracted from the integrated single-cell transcriptomic data. Following data normalization, selection of highly variable genes, and batch effect correction, dimensionality reduction and visualization were performed using the UMAP algorithm. Cell trajectory analysis was conducted using the monocle3 (v1.4.26) package. For B-cell subsets, “naive B cells” were designated as the differentiation starting point. For T-cell subsets, including MAIT and CD8^+^ T cells, no starting point was assigned. Pseudo-time values were calculated for each cell along the differentiation trajectories. A graph-based statistical test in monocle3 was then applied to identify DEGs that varied significantly along the trajectories. Finally, the gene expression visualization function in monocle3 was used to display the dynamic expression patterns of key regulatory genes during the differentiation process.

### 2.11 BCR/TCR analysis

TCR/BCR sequencing data were preprocessed using the Cell Ranger VDJ pipeline (v7.1.0) and subjected to quality control. Subsequent immune repertoire analysis was performed with the immunarch (v 0.10.3) and scRepertoire (v 2.4.0) packages. The files processed by Cell Ranger for BCR and TCR were separately loaded. The annotation files for BCR and TCR were then stored in independent lists respectively. The BCR and TCR data from multiple samples were integrated into a single object using the combineBCR and combineTCR functions, respectively, with the clonal similarity threshold set to 0.85 in BCR. The addVariable function was then used to append grouping variables to the integrated data object. A series of quantitative and comparative analyses were performed on the integrated repertoire data. The proportion and absolute count of cells with productive rearrangements in each sample were calculated and visualized using the clonalQuant function. The clonal proportion within samples was analyzed via the clonalProportion function. High-frequency clonotypes shared between different groups were identified and visualized employing the clonalCompare function.

### 2.12 Flow cytometry analysis

The proportions of CD8^+^ T cells and mucosal-associated invariant T (MAIT) cells in human peripheral blood samples were analyzed by flow cytometry. Whole blood was collected using 2 ml EDTA vacutainer. A 100 μL aliquot of fresh whole blood was incubated with a pre-mixed antibody cocktail (containing fluorescently labeled monoclonal antibodies against human CD3, CD8, TCR Vα7.2, and CD161, each at a 1:100 dilution) for 15 minutes at room temperature in the dark. Subsequently, 2 mL of red blood cell lysis buffer was added, followed by incubation for 5 minutes at room temperature to lyse erythrocytes. The cells were then centrifuged (room temperature, 500 × g, 5 minutes), and the supernatant was discarded. If lysis was incomplete, the lysis step was repeated. The cell pellet was washed once with 1 mL of PBS by gentle pipetting, centrifuged again (500 × g, 5 minutes), and the supernatant was removed. Finally, the cells were resuspended in 200 μL of PBS and acquired on a BD LSRFortessa flow cytometer. Data analysis was performed using FlowJo software. CD8^+^ T cells (identified as CD3^+^CD8^+^) and MAIT cells (defined as CD3^+^TCR Vα7.2^+^CD161^+^) were gated and their frequencies were calculated as a percentage of CD3^+^ T cells.

### 2.13 qPCR

The expression level of *IFI44L* in PBMCs was analyzed by RT-qPCR. PBMCs were obtained using the same method as described for scRNA-seq and lysed in TRIzol reagent. The chloroform was added for phase separation. After centrifugation, the aqueous phase was collected, and RNA was precipitated by adding 500 μL of isopropanol. The mixture was gently inverted for 15 seconds and centrifuged at 12,000 × g for 10 min at 4 °C. The supernatant was discarded, and the RNA pellet was then washed twice with 75% ethanol, air-dried at room temperature for 3 min, and dissolved in 15 μL DEPC-treated water. RNA concentration and the A260/A280 and A260/230 ratios were measured with a Nanodrop spectrophotometer. A 20 μL reverse transcription reaction mixture was prepared and heated to synthesize cDNA according to the manufacturer’s instructions. The resulting cDNA was diluted and used to prepare a 20 μL qPCR mix as directed by the manufacturer, containing SYBR Green Real-Time PCR Master Mix, cDNA, primers, and nuclease-free water. Quantitative PCR began with a 60 s denaturation step at 95 °C, followed by 40 cycles of 95 °C for 15 s (denaturation), 60 °C for 15 s (annealing), and 72 °C for 45 s (extension). After the run, Ct values of target genes and the internal control GAPDH were recorded. Relative gene expression was calculated by the 2^-ΔΔCt^ method for normalization.

### 2.14 Statistical analysis

Differential gene expression analysis for cell subsets was performed as described in Section 2.7. For flow cytometry and qPCR data, differences between groups were assessed using an independent two-sample t-test. All statistical analyses were conducted with R and SPSS. A *P*-value of less than 0.05 was considered statistically significant.

## 3 Result

### 3.1 Analysis of cell type composition in peripheral blood single-cell data

**Figure 1:**
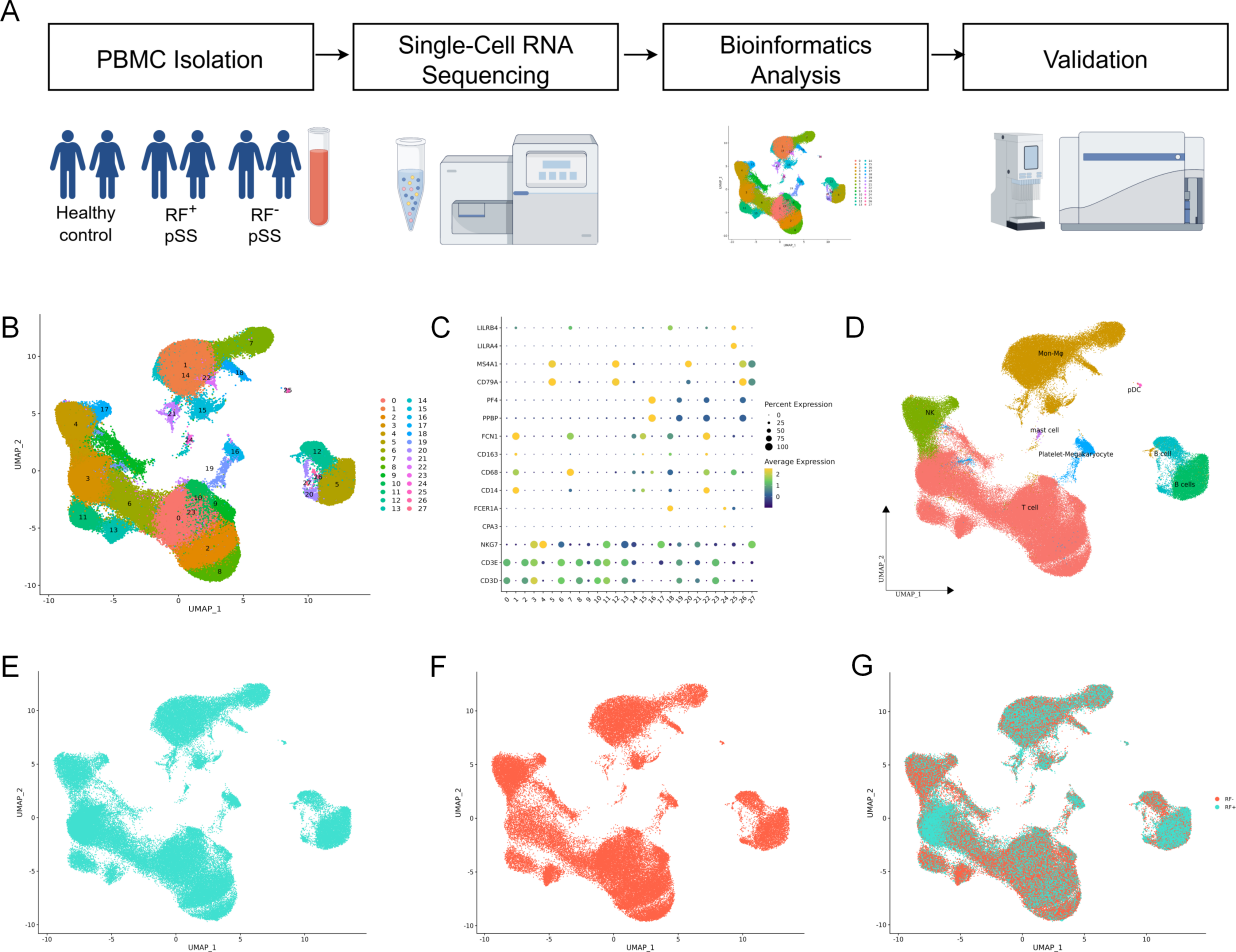
PBMCs cellular landscape and compositional patterns. **(A)** Schematic diagram of the research process. **(B)** UMAP visualization depicting cell embeddings for all participants. **(C)** Dot plot illustrating the gene expression characteristics across distinct cell populations for all participants. **(D)** Annotated UMAP plot following cell type classification based on identified marker genes. **(E)** UMAP visualization of PBMCs from the RF^+^ group. **(F)** UMAP visualization of PBMCs from the RF^-^ group. **(G)** UMAP plot showing intermixed distribution of RF^+^ and RF^-^ groups across clusters.

The clinical information for all participants is summarized in **Table 2** and **Supplementary Table 1**. scRNA-seq was performed on their PBMCs, followed by downstream analysis (**Figure 1A**). A total of 172,066 PBMCs were captured, with 77,496 from the pSS RF^+^ group, 59,202 from the pSS RF^-^ group, and 35,368 from the HC group. Unsupervised clustering of all cells identified 28 distinct clusters, which were visualized via UMAP (**Figure 1B**). Clusters 0, 2, 3, 6, 8, 9, 10, 11, 13, and 23 were annotated as T cells based on the expression of the marker gene *CD3*; clusters 5, 12, 20, 26, and 27 were defined as B cells based on the expression of marker genes *CD79A* and *MS4A1*; clusters 4 and 17 were identified as NK cells due to the high expression of *NKG7* and low expression of *CD3*; clusters 1, 7, 14, 15, 18, 21, and 22 were defined as monocytes-macrophages (Mon-Mφ) given the expression of *CD14*, *CD68*, *CD163*, and *FCN1*; clusters 16 and 19 were designated as platelet-megakaryocytes owing to the expression of *PPBP* and *PF4*; cluster 25 was defined as pDCs based on the expression of *LILRA4* and *LILRB4*; and cluster 24 was identified as mast cells due to the expression of *CPA3* and *FCER1A* (**Figure 1C**). The UMAP was subsequently re-displayed according to the annotation results (**Figure 1D**). Furthermore, PBMCs from RF^+^ group (**Figure 1E**) and RF^-^ group (**Figure 1F**) were extracted and visualized separately via UMAP, and the two groups were also merged for combined visualization (**Figure 1G**).

**Table 2:** Clinical characteristics of all participants.

Characteristics	HC (N = 3)	RF^+^ pSS (N = 6)	RF^-^ pSS (N = 6)	*P*-value (RF^+^ vs. RF^-^)
Age, years	42.67 ± 6.34	58.50 ± 9.01	47.67 ± 13.59	0.106
Female, n (%)	3 (100%)	6(100%)	6(100%)	1.000
Disease duration, years		5.17 ± 5.04	10.67 ± 12.26	0.345
WBC (× 10^9^/L)		4.21 ± 2.32	4.32 ± 1.63	0.924
NEUT (× 10^9^/L)		2.46 ± 1.40	2.29 ± 0.82	0.799
LYM (× 10^9^/L)		1.30 ± 0.75	1.38 ± 0.65	0.841
PLT (× 10^9^/L)		196.33 ± 73.44	209.67 ± 57.78	0.734
IgG (g/L)		22.45 ± 6.77	13.11 ± 4.06	0.016*
IgA (g/L)		3.90 ± 1.29	2.99 ± 1.39	0.269
IgM (g/L)		1.41 ± 0.69	1.04 ± 0.49	0.304
C3 (g/L)		0.79 ± 0.19	0.81 ± 0.26	0.872
C4 (g/L)		0.17 ± 0.01	0.23 ± 0.12	0.262
CRP (mg/dL)		0.19 ± 0.05	0.19 ± 0.22	0.918
ESR (mm/h)		34.33 ± 15.12	9.83 ± 8.37	0.006*
ESSDAI		10.00 ± 8.00	4.50 ± 2.59	0.160

WBC, white blood cell count; NEUT, neutrophil count; LYM, lymphocyte count; PLT, platelet count; IgG, immunoglobulin G; IgA, immunoglobulin A; IgM, immunoglobulin M; CRP, C-reactive protein; ESR, erythrocyte sedimentation rate; ESSDAI, the European league against Rheumatism classification criteria (EULAR) Sjögren’s syndrome disease activity index. *P < 0.05.

### 3.2 Characterization of B and T cell subsets

Given the potential disparities in the proportion or function of specific T and B cell subsets associated with RF status in pSS, T and B cell populations were isolated and re-clustered to resolve their subset architecture. Each subset was annotated based on its most highly expressed characteristic marker genes.

**Figure 2:**
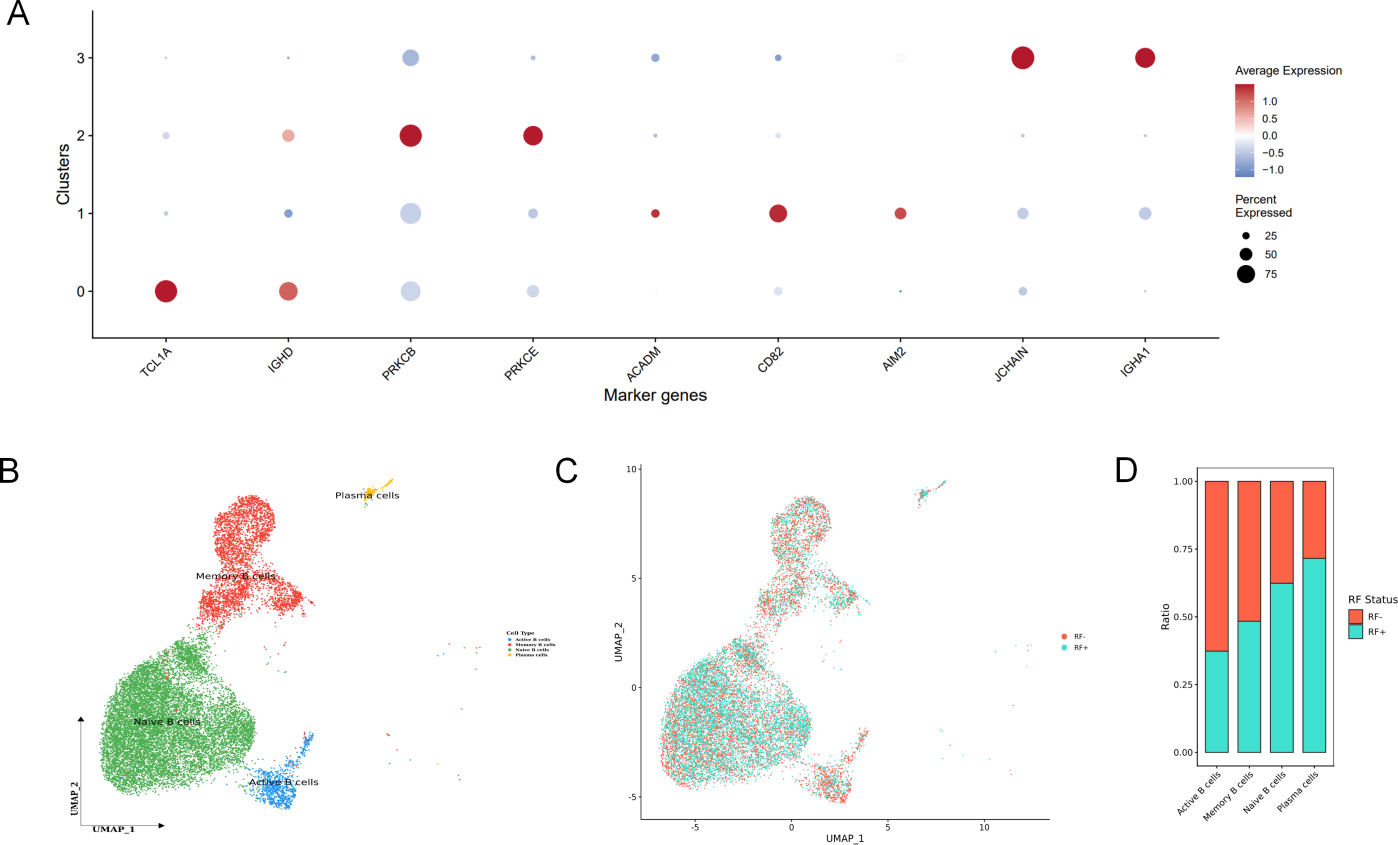
Analysis of B cell subsets in RF^+^ and RF^-^ groups. **(A)** Dot plot showing gene expression signatures across distinct B cell subpopulations. **(B)** Annotation of B cell subpopulations on the UMAP. **(C)** UMAP plot illustrating the intermixed distribution of RF^+^ and RF^-^ cells across B cell clusters. **(D)** Bar chart showing the proportional composition of RF^+^ and RF^-^ groups within each B cell subset.

Based on the gene expression profiles (**Figure 2A**), B cells were further clustered and identified into 4 subsets: naïve B cells, memory B cells, active B cells and plasma cells (**Figure 2B**). UMAP visualization revealed a notable difference in local cell density was observed specifically within the plasma cell cluster between the two groups (**Figure 2C**). Quantification of the proportion of RF^+^ and RF^-^ cells within each B cell subset further confirmed this observation, showing a significantly higher percentage of plasma cells in the RF^+^ group compared to the RF^-^ group (**Figure 2D**).

**Figure 3:**
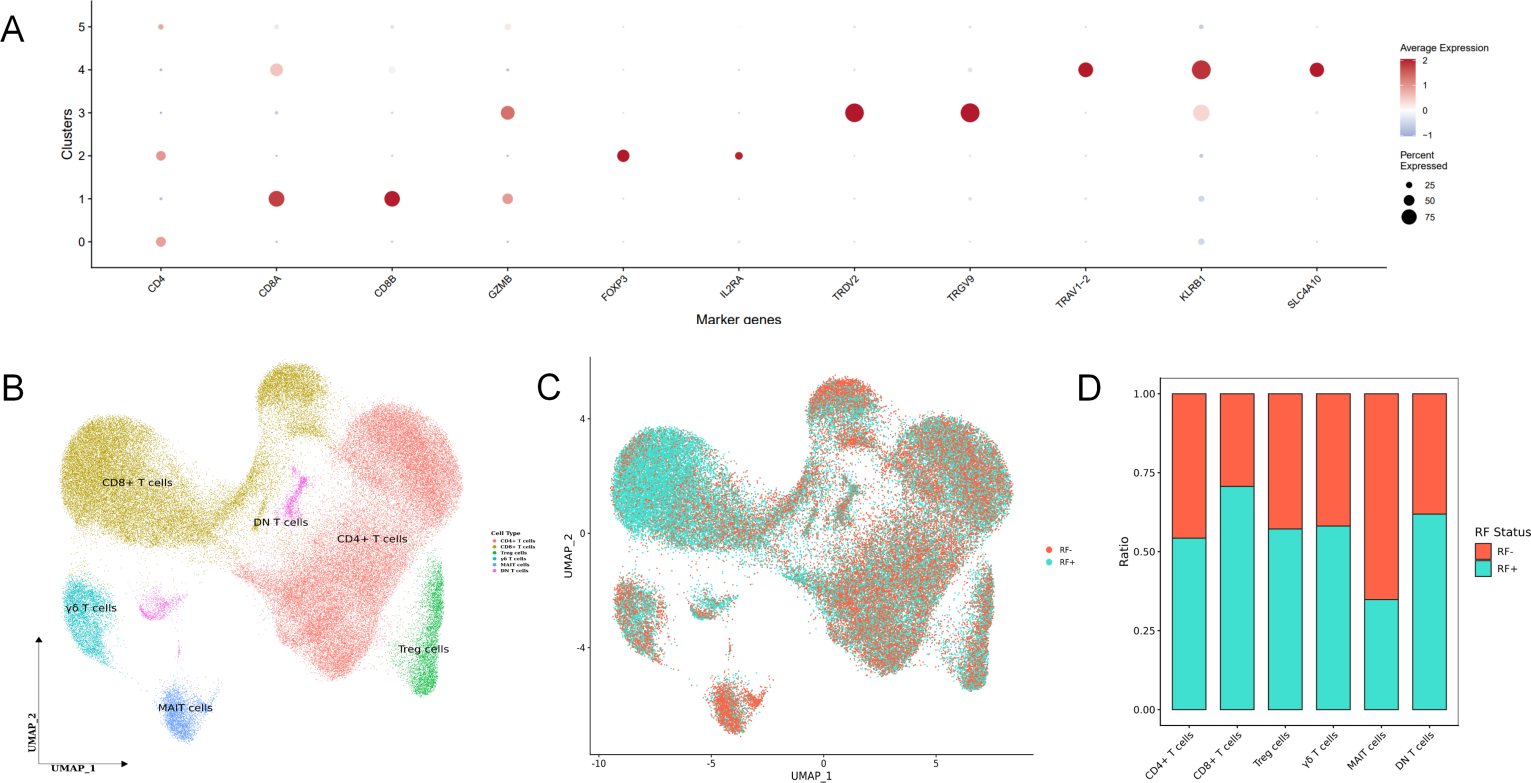
Analysis of T cell subsets in RF^+^ and RF^-^ groups. **(A)** Dot plot showing gene expression signatures across distinct T cell subpopulations. **(B)** Annotation of T cell subpopulations on the UMAP. **(C)** UMAP plot illustrating the intermixed distribution of RF^+^ and RF^-^ cells across T cell clusters. **(D)** Bar chart showing the proportional composition of RF^+^ and RF^-^ groups within each T cell subset.

Based on the gene expression profiles (**Figure 3A**), T cells were further subdivided into 6 subsets, including CD4^+^ T cells, CD8^+^ T cells, Tregs, Double- negative (DN) T cells, γδT cells and MAIT cells (**Figure 3B**). The UMAP plot showed an intermixed distribution of RF^+^ and RF^-^ cells across T cell clusters, with apparent differences in local density within the CD8^+^ T cell and MAIT cell clusters (**Figure 3C**). Proportional analysis confirmed that the RF^+^ group had a significantly higher proportion of CD8^+^ T cells and a significantly lower proportion of MAIT cells compared to the RF^-^ group (**Figure 3D**).

### 3.3 Differential gene comparison and functional enrichment analysis

**Figure 4:**
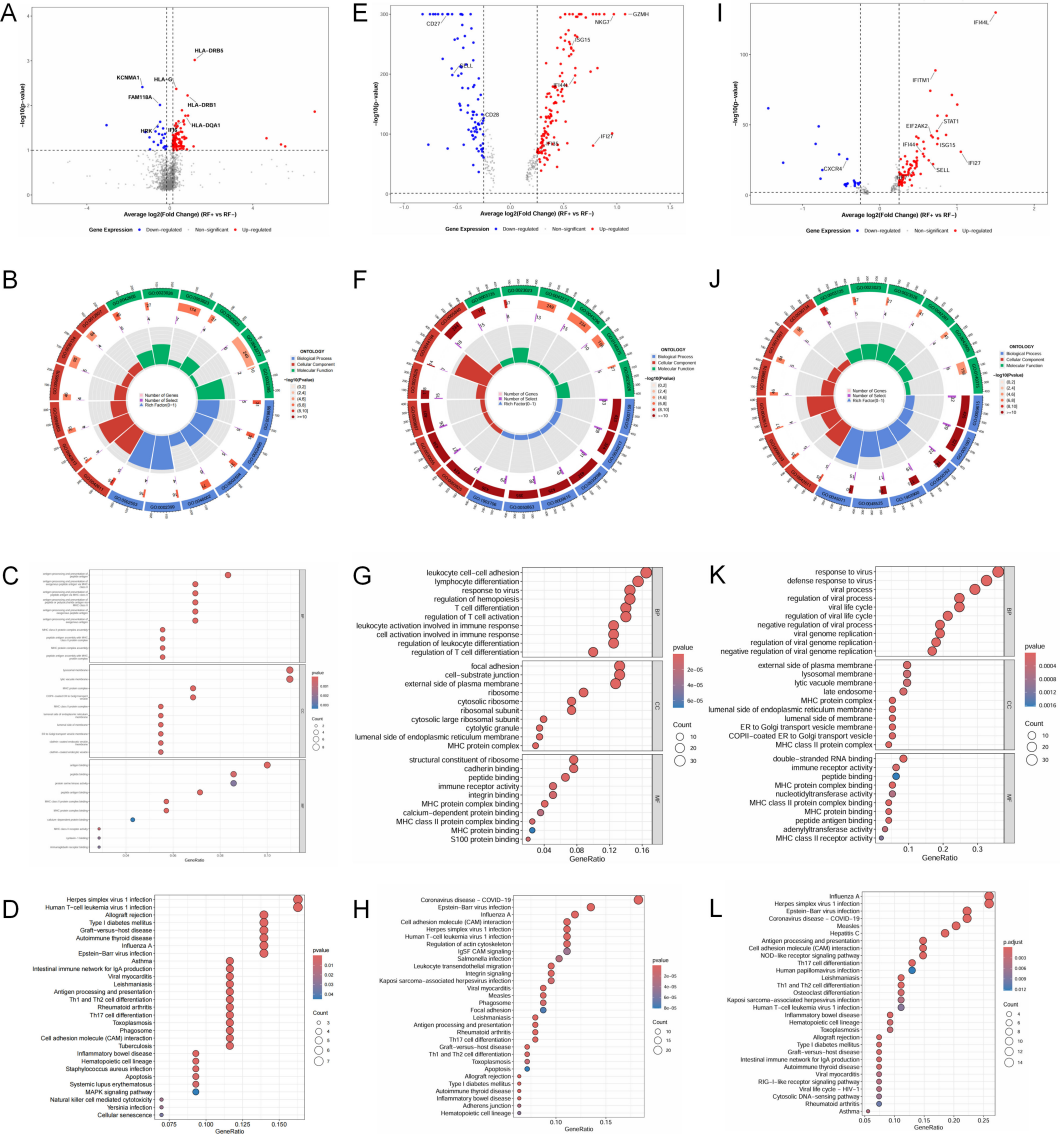
Differential gene identification and functional enrichment analysis. **(A)** Volcano plot of differentially expressed genes in plasma cells between RF^-^ and RF^+^ groups. **(B)** Donut plot of GO enrichment analysis for plasma cells. **(C)** Bubble plot of GO enrichment analysis for plasma cells. **(D)** Bubble plot of KEGG pathway enrichment analysis for plasma cells. **(E)** Volcano plot of differentially expressed genes in CD8^+^ T cells between RF^-^ and RF^+^ groups. **(F)** Donut plot of GO enrichment analysis for CD8^+^ T cells. **(G)** Bubble plot of GO enrichment analysis for CD8^+^ T cells. **(H)** Bubble plot of KEGG pathway enrichment analysis for CD8^+^ T cells. **(I)** Volcano plot of differentially expressed genes in MAIT cells between RF^-^ and RF^+^ groups. **(J)** Donut plot of GO enrichment analysis for MAIT cells. **(K)** Bubble plot of GO enrichment analysis for MAIT cells. **(L)** Bubble plot of KEGG pathway enrichment analysis for MAIT cells.

Subset analysis revealed significant differences in the distribution of plasma cells, CD8^+^ T cells, and MAIT cells between the RF^+^ and RF^-^ groups. To further elucidate their functional heterogeneity, differential gene expression analysis and functional enrichment studies were subsequently performed on these subsets. A total of 82 DEGs were identified in the plasma cell subpopulation between RF^+^ and RF^-^ groups, among which 55 genes were significantly up-regulated and 27 genes were significantly down-regulated in RF^+^ patients. The upregulated genes include antibody-related genes such as *IGLV2–18* and *IGHV3-74*, as well as interferon-stimulated genes including *IFI6*, *LY6E*, *BST2*, *IFITM2*, and *ZFP36L1*. These findings indicate enhanced clonal expansion, antibody production, and immune activation in plasma cells from RF^+^ patients (**Figure 4A**). Unexpectedly, antigen presentation and immunomodulation-related genes, such as *HLA-DRB1*, *HLA-DRB5*, *HLA-DQA1*, *HLA-DMA*, and *HLA-G*, also exhibited significant upregulation. Based on GO enrichment analysis, the DEGs identified were primarily involved in a series of events related to antigen processing and presentation (**Figures 4B, C**). KEGG pathway enrichment analysis demonstrated that the DEGs were significantly enriched in multiple pathways closely associated with autoimmunity and inflammatory responses. Moreover, pathways related to immune cell differentiation and immune responses to specific pathogens were also notably enriched (**Figure 4D**).

Comparison between the RF^+^ and RF^-^ groups identified 220 DEGs in CD8^+^ T cells, comprising 138 upregulated and 82 downregulated genes. The upregulated genes primarily included core effector molecules of the interferon response, such as *ISG15*, *IFI27*, *IFI44L*, *IFI6*, and *IFI35*, as well as cytotoxic effector molecules including *NKG7*, *GNLY*, *PRF1*, *GZMA*, *GZMB*, and *GZMH*. The downregulated genes involved costimulatory and activation receptors, including *CD28*, *CD27*, and *SELL*. These findings suggest that CD8^+^ T cells from RF^+^ patients exhibit features of strong immune activation, enhanced cytotoxicity, and hyperactive interferon signaling (**Figure 4E**). GO enrichment analysis revealed that the DEGs were significantly enriched in terms related to immune activation and effector functions. Within the Biological Process category, genes were predominantly enriched in processes involving leukocyte/lymphocyte activation and differentiation, such as leukocyte cell-cell adhesion and T cell differentiation, as well as the response to virus. Cellular Component analysis indicated that genes were significantly localized to structures associated with immune synapses and cytotoxicity, including the external side of plasma membrane, MHC protein complex, and cytolytic granule. At the Molecular Function level, the enrichment highlighted prominent roles in immune receptor activity and the capacity for MHC protein/peptide binding (**Figures 4F, G**). KEGG pathway enrichment analysis revealed that the DEGs were significantly enriched in pathways related to viral infection and immune response, immune cell migration and activation and autoimmunity and inflammation pathways (**Figure 4H**).

For MAIT cells, a total of 105 DEGs were identified, with 82 genes up-regulated and 23 genes down-regulated in the RF^+^ group (**Figure 4I**). The up-regulated gene set included numerous classical interferon-stimulated genes, such as *ISG15*, *IFI27*, *IFI44L*, *IFI44*, *IFI6*, *IFI16*, *IFIT3*, and *IFITM1/2/3*, as well as key regulators of interferon signaling pathways, including *STAT1*, *IRF7*, and *EIF2AK2*. Down-regulation of *CXCR4* and *SELL* may promote the infiltration and retention of MAIT cells from peripheral blood into local tissues such as glands, thereby contributing directly to localized tissue damage. This observation aligns with the lower proportion of MAIT cells found in the peripheral blood of RF^+^ individuals compared with RF^-^ subjects. GO analysis revealed that DEGs were enriched in biological processes related to viral response and regulation. For cellular components, enrichments occurred in membrane structures and immune complexes. In molecular functions, the genes were primarily enriched in immune-associated binding activities (**Figures 4J, K**). KEGG enrichment analysis results showed that the DEGs were significantly enriched primarily in antiviral immunity-related pathways and immune system and inflammatory disease pathways (**Figure 4L**).

### 3.4 Cell communication network analysis

**Figure 5:**
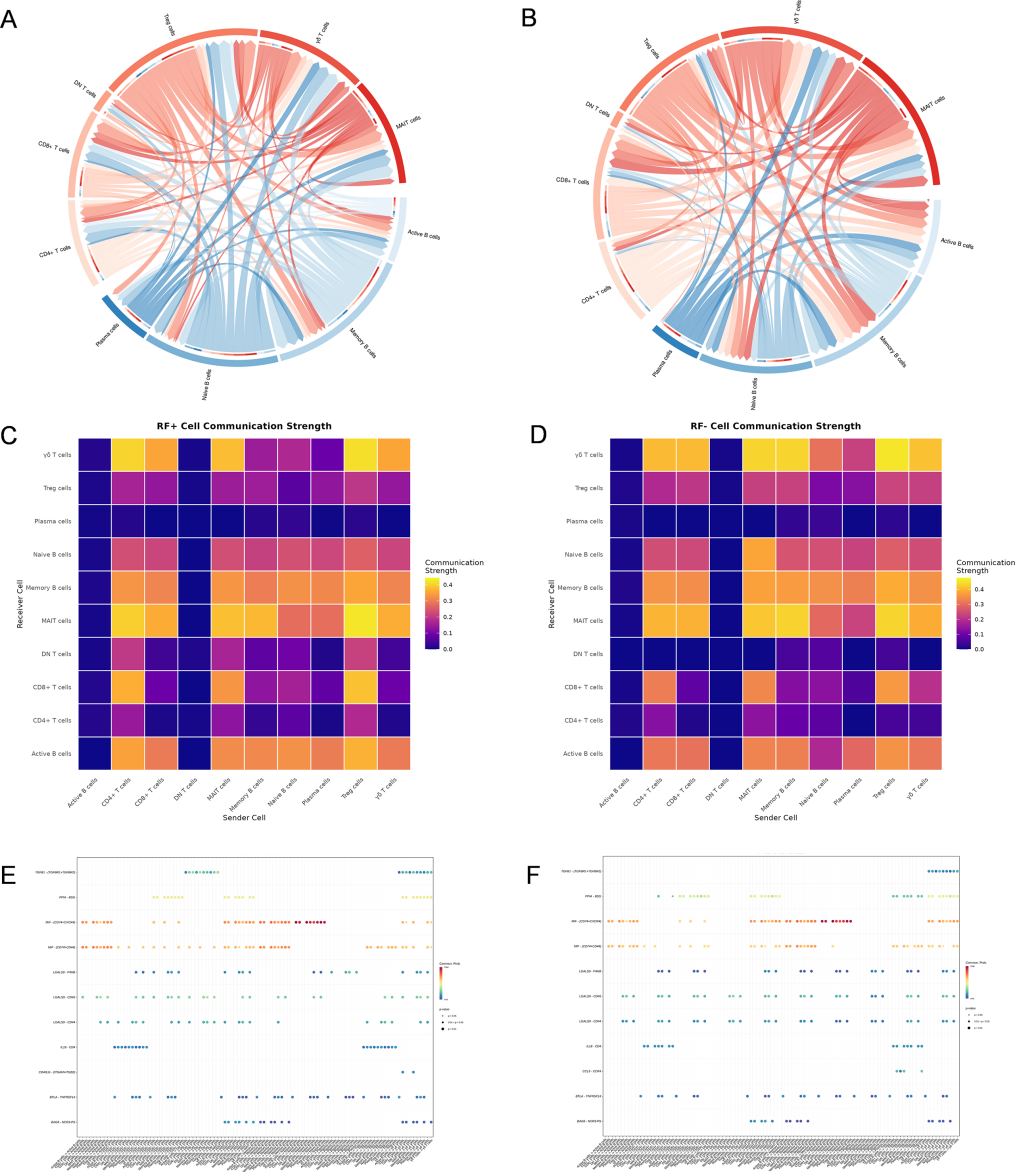
Inference of cell–cell communication by CellChat. **(A)** Circle plots depicting the inferred number of interactions **(B)** Circle plots depicting the overall strength of interactions. **(C)** Heatmaps showing the strength of intercellular communication networks in the RF^+^ groups. **(D)** Heatmaps showing the strength of intercellular communication networks in the RF^-^ groups. **(E)** Heatmaps showing the counts of significant ligand–receptor pairs contributing to the cell–cell communication in the RF^+^ groups. **(F)** Heatmaps showing the counts of significant ligand–receptor pairs contributing to the cell–cell communication in the RF^-^ groups.

To investigate the effect of RF status on cell-cell communication in the immune microenvironment, CellChat was used to computationally infer the number and strength of intercellular interactions across all groups (**Figures 5A, B**). Further comparative analysis between the RF^+^ and RF^-^ groups indicated that the overall communication strength was higher in the RF^+^ group. Tregs were predicted to exhibit the strongest signaling output toward nearly all cell types, while the MAIT cells were inferred to be important signal receivers in RF+ and RF- groups (**Figures 5C, D**). CellChat-based ligand-receptor analysis predicted that the *MIF*-(*CD74*+*CXCR4*/*CD44*) signaling pathway was the dominant computationally inferred pathway mediating T–B cell collaboration in both groups (**Figures 5E, F**).

### 3.5 Pseudo-time analysis of B and T cell subtypes

**Figure 6:**
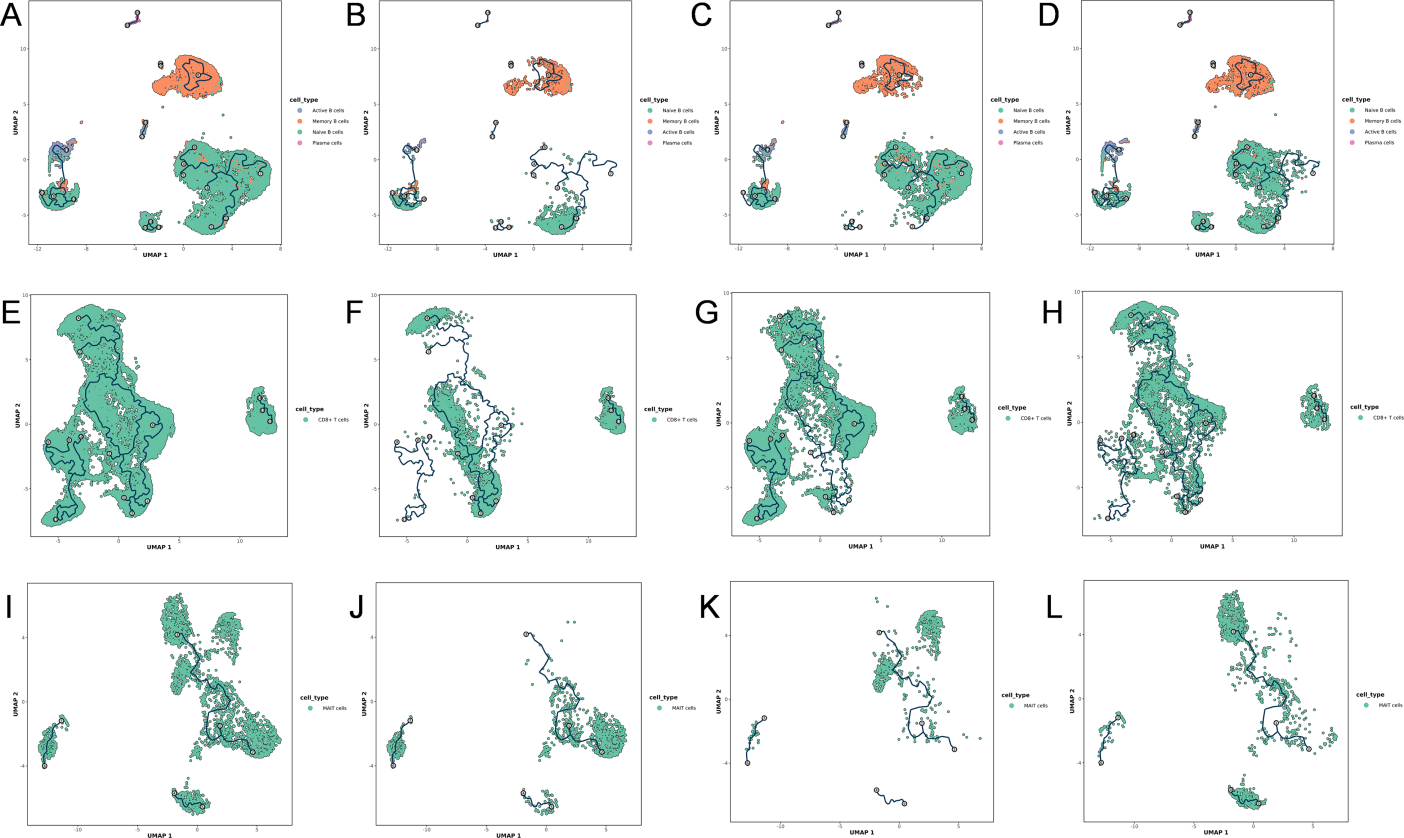
Pseudotime trajectory analysis of B cell, CD8^+^ T cell, and MAIT cell differentiation. **(A)** B cell differentiation trajectory across all groups. **(B)** B cell trajectories in the HC group. **(C)** B cell trajectories in the RF^+^ group. **(D)** B cell trajectories in the RF^-^ group. **(E)** CD8^+^ T cell differentiation trajectory across all groups. **(F)** CD8^+^ T cell trajectories in the HC group. **(G)** CD8^+^ T cell trajectories in the RF^+^ group. **(H)** CD8^+^ T cell trajectories in the RF^-^ group. **(I)** MAIT cell differentiation trajectory across all groups. **(J)** MAIT cell trajectories in the HC group. **(K)** MAIT cell trajectories in the RF^+^ group. **(L)** MAIT cell trajectories in the RF^-^ group.

Further trajectory analysis was performed to computationally infer putative differentiation states of B and T cells. Although it does not capture real-time differentiation, pseudo-time analysis reconstructs developmental trajectories by ordering cells according to transcriptomic similarity. The pseudo-time trajectory of B cells illustrates a pathway starting from naïve B cells, proceeding through activated B cells, and ultimately differentiating into either memory B cells or plasma cells (**Figure 6A**). Both RF^+^ and RF^-^ groups exhibited distinct cellular developmental trajectories compared to the HC group (**Figures 6B–D**). For naïve B cells, the HC group was predominantly distributed in the early stage of the trajectory, the RF^-^ group was concentrated in the early-to-mid stage, and the RF^+^ group was mainly located in the mid-to-late stage. In terms of memory B cells, the RF^+^ group showed a more complete differentiation trajectory than the RF^-^ and HC groups. For activated B cells, the RF^-^ group was primarily clustered in the early phase of the differentiation trajectory, whereas the RF^+^ group tended to localize more toward the mid-to-late stage. Regarding plasma cells, the HC group had a sparse distribution, and the RF^+^ group was more concentrated in the early differentiation stage compared to the RF^-^ group. Overall, the inferred pseudo-time ordering suggests that B cell subsets in RF^+^ patients may be skewed toward later stages of a computationally reconstructed differentiation trajectory, which is consistent with B cell immune dysregulation. The distribution and differentiation patterns of B cells in RF^-^ patients were intermediate between those of the HC group and the RF^+^ group. Similarly, for CD8^+^ T cells, both the RF^+^ and RF^-^ groups exhibited cellular developmental trajectories distinct from those of the HC group (**Figure 6E**). CD8^+^ T cells from the HC group were predominantly distributed in the early and terminal phases of the trajectory (**Figure 6F**), whereas the RF^+^ group was mainly concentrated in the mid-to-late stage (**Figure 6G**). The distribution pattern of the RF^-^ group lay between that of the HC and RF^+^ groups (**Figure 6H**). The developmental pattern of MAIT cells also differed among the 3 groups (**Figure 6I**). The HC group was predominantly concentrated in the early and terminal phases of the trajectory (**Figure 6J**). The RF^+^ group exhibited a relatively dispersed trajectory with noticeable reduction in both early and late stages (**Figure 6K**), while the RF^-^ group was mainly located in the mid-phase of the trajectory (**Figure 6L**). As an autoimmune disease, pSS is characterized by significant immune imbalance in patients. Pseudo-time analysis further suggests that RF^+^ and RF^-^ pSS patients may display distinct patterns along computationally inferred differentiation trajectories, which could serve as a hypothesis-generating meaningful basis for patient stratification.

### 3.6 BCR/TCR analysis

**Figure 7:**
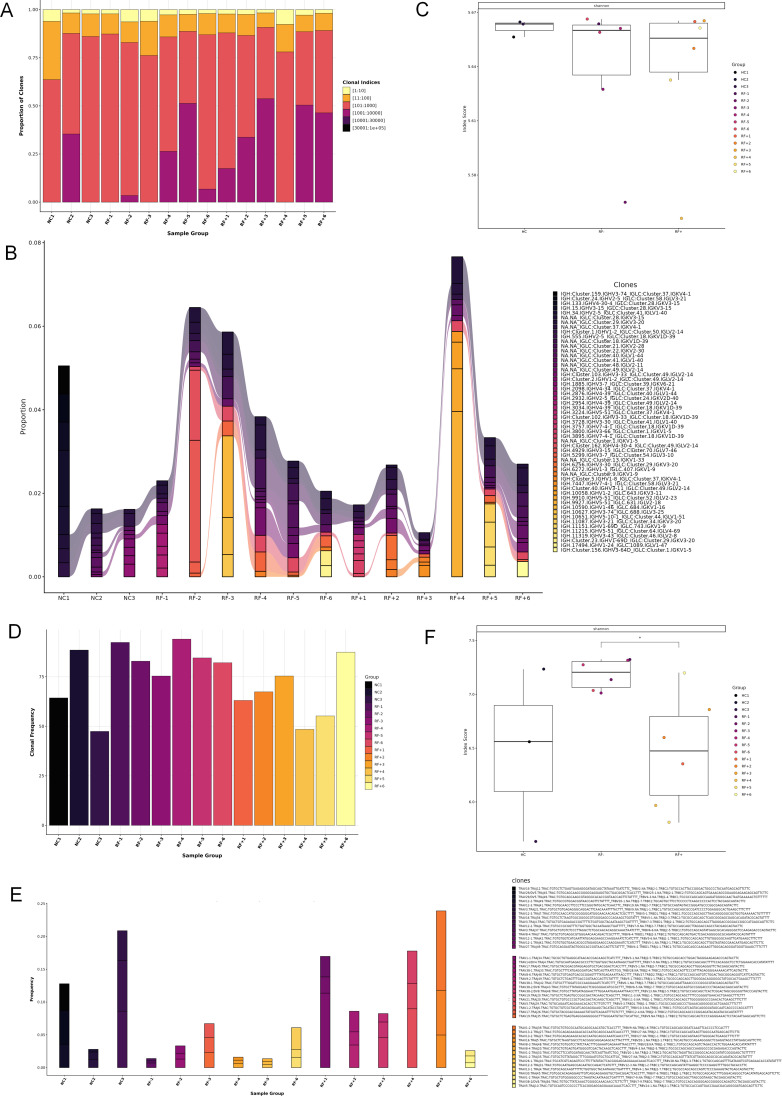
Comparative analysis of BCR and TCR repertoires between HC, RF^+^ and RF^-^ pSS groups. **(A)** Composition of the BCR repertoire based on clonal size distribution. **(B)** Network of BCR clonal sharing across all samples. **(C)** Box plot of the BCR clone diversity comparison evaluated by Shannon index. **(D)** Composition of the TCR repertoire based on clonal frequency. **(E)** Patterns of TCR clonal sharing across all samples. **(F)** Box plot of the TCR clone diversity comparison evaluated by Shannon index. ∗*P* < 0.05.

Clonotype proportion analysis revealed differences in the distribution of BCR clonotypes between the RF⁺ and RF⁻ groups. In the RF⁺ group, clonotypes ranked 1,001–10,000 by abundance accounted for a larger proportion of the repertoire than those in the RF⁻ group (**Figure 7A**). This finding suggests that the BCR repertoire in the RF⁺ group exhibits a more pronounced long-tail distribution, with a greater contribution from relatively low-abundance clonotypes to the immune response, potentially reflecting broader B-cell activation. Further analysis of dominant BCR clonotypes demonstrated marked differences in clonotype composition among individual samples. Some clonotypes were detected across multiple samples, indicating a certain degree of shared BCR clonotypes among individuals. Meanwhile, prominent expansion of dominant clonotypes was observed in selected samples, suggesting substantial inter-individual heterogeneity in BCR clonal expansion (**Figure 7B**). To further evaluate the overall diversity of the BCR repertoire across groups, we compared the Shannon diversity index. No statistically significant differences were observed among the HC, RF⁻, and RF⁺ groups (**Figure 7C**). These results indicate that, although the RF⁺ and RF⁻ groups differ in clonotype abundance distribution and dominant clonotype composition, the overall clonal diversity of the BCR repertoire remains comparable across groups. TRA-chain clonal frequency analysis revealed marked differences in T-cell clonotype composition among individual samples. The RF⁻ group generally exhibited higher TRA-chain clonal frequencies, whereas the RF⁺ group showed more pronounced inter-individual heterogeneity (**Figure 7D**). Different patients exhibited distinct dominant clonotype profiles, suggesting individualized characteristics of T-cell immune responses (**Figure 7E**). Shannon diversity index analysis revealed that TCR repertoire diversity differed significantly between the RF^+^ and RF ^-^ groups (**Figure 7F**), with the RF ^-^ group exhibiting higher clonal diversity compared to the RF^+^ group. These findings suggest that the more restricted TCR diversity in RF^+^ patients reflects a more focused, antigen-driven T-cell response.

### 3.7 Flow cytometry and qPCR analysis

**Figure 8:**
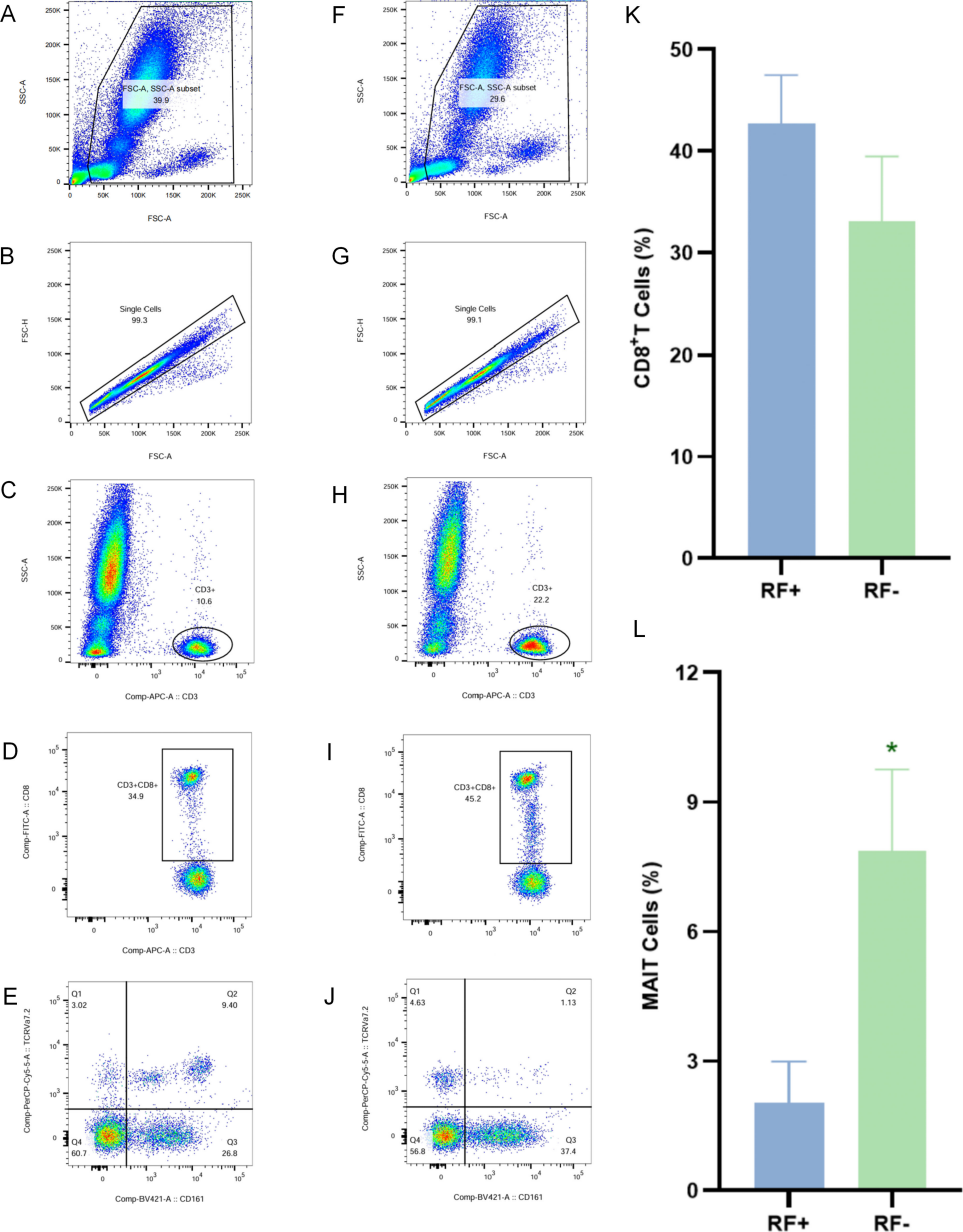
Flow cytometry and qPCR analyses. **(A)** Viability gating (live/dead discrimination) in the RF^+^ group. **(B)** Singlet gating (doublet exclusion) in the RF^+^ group. **(C)** Proportion of CD3^+^ T cells within lymphocytes in the RF^+^ group. **(D)** Proportion of CD8^+^ T cells within CD3^+^ T cells in the RF^+^ group. **(E)** Proportion of MAIT cells (CD3^+^TCR Vα7.2^+^CD161^+^) within CD3^+^ T cells in the RF^+^ group. **(F)** Viability gating (live/dead discrimination) in the RF^-^ group. **(G)** Singlet gating (doublet exclusion) in the RF^-^ group. **(H)** Proportion of CD3^+^ T cells within lymphocytes in the RF^-^ group. **(I)** Proportion of CD8^+^ T cells within CD3^+^ T cells in the RF^-^ group. **(J)** Proportion of MAIT cells (CD3^+^TCR Vα7.2^+^CD161^+^) within CD3^+^ T cells in the RF^-^ group. **(K)** Frequency of CD8^+^ T cells in PBMCs between RF^-^ and RF^+^ groups. **(L)** Frequency of MAIT cells in PBMCs between RF^-^ and RF^+^ groups. **(M)** Relative mRNA expression of *IFI44L* in PBMCs between RF^-^ and RF^+^ groups. Data are presented as mean ± SEM; n = 6 biological replicates. ∗*P* < 0.05 compared with the RF^+^ group.

The proportions of CD8^+^ T cells and MAIT cells in peripheral blood were measured by flow cytometry in patients with pSS stratified by RF status (**Figures 8A–J**). Compared with the RF^-^ group, RF^+^ patients showed a significant increase in the frequency of CD8^+^ T cells (**Figure 8K**) and a decrease in the frequency of MAIT cells (**Figure 8L**). These differences were statistically significant, and the findings are consistent with the scRNA-seq analysis of the corresponding cellular subsets. Given the consistent upregulation of interferon-stimulated genes (ISGs) observed in both MAIT cells and CD8^+^ T cells from RF^+^ patients, we selected *IFI44L*, which was elevated in both subsets for validation by qPCR in PBMCs. This analysis confirmed the upregulation of *IFI44L* in RF^+^ patients compared to RF^-^ patients (**Figure 8M**).

## 4 Discussion

RF is an autoantibody that targets the Fc segment of denatured IgG and is extensively found in patients with autoimmune diseases (1, 22–25). Studies have shown that RF has been reported with a prevalence ranging from 40% to 90% in patients with pSS (1, 2, 26, 27) and RF is an independent risk factor for the development of lymphoma in pSS (10, 11). RF and its subtypes play a critical role in the diagnosis of pSS (28). Previous studies by our team have demonstrated that with increasing RF titers, the incidence of hematologic involvement and arthritis in pSS patients shows an upward trend, accompanied by a more active immune-inflammatory response (27, 29). Furthermore, aberrant activation of B cells is considered the central mechanism underlying the development of pSS (30–32). A variety of cytokines and immune cells stimulate aberrant B-cell maturation, leading to the production of a spectrum of autoantibodies. Targeted intervention against aberrantly activated B cells is regarded as a promising therapeutic approach for pSS, although the effectiveness demonstrated in randomized controlled trials remains a subject of debate (33–35). Denatured IgG with its Fc fragment serving as an autoantigen, is taken up, processed, and presented by antigen-presenting cells. With the help of specific T cells, B cells are activated and differentiated into plasma cells, thereby producing autoantibodies such as RF targeting the Fc fragment of IgG. In summary, B cells play a pivotal role in both the disease progression of pSS and the production of RF.

Accordingly, this study first compared B-cell subset changes between RF^+^ and RF^-^ pSS patients. We observed significantly higher proportions of plasma cells and naïve B cells in RF^+^ patients. Further screening of DEGs in plasma cells from the two patient groups and functional enrichment analysis revealed that plasma cells from RF^+^ and RF^-^ pSS patients not only differed in proportion but also exhibited significant differences in functional status. Plasma cells from RF^+^ patients displayed a highly activated state, with up-regulated genes indicating stronger antibody production and secretion capacity, which was closely associated with type I interferon activation. This finding aligns with the RF-positive phenotype and the clinical observation that RF^+^ patients have higher serum IgG levels than RF^-^ patients. The further activation of the core pathological mechanism of pSS, the type I interferon pathway, may contribute to the more active immune-inflammatory response observed in RF^+^ patients (36). Trutschel et al. (37) integrated multi-omics data and identified IFNα as a key cytokine driving disease heterogeneity in pSS. Similarly, Zhang et al. (38) through scRNA-seq, demonstrated that B cells participate in the pathogenesis of pSS via the type I interferon signaling pathway. Meanwhile, significant upregulation of antigen processing and presentation-related genes was also observed in plasma cells from RF^+^ patients. This phenomenon may be related to the promotion of HLA molecule expression by type I interferon (39, 40). Analysis of BCR sequencing revealed that the RF⁺ group showed a more pronounced long-tail distribution of BCR clonotypes, though Shannon diversity index analysis did not reveal statistically significant differences in overall BCR clonal diversity among groups. These findings are consistent with the highly activated functional state of plasma cells observed in the differential expression analysis, and together suggest that the B-cell immune response in RF^+^ pSS patients is characterized by broader B-cell activation and heterogeneous clonal expansion. Mechanistically, this pattern is compatible with chronic antigenic stimulation driving iterative rounds of B cell selection and affinity maturation within germinal centers, thereby contributing to the emergence of shared or individually expanded autoreactive BCR clonotypes. This interpretation is supported by the proteogenomic analysis of the autoreactive BCR in pSS patients by Broeren et al. (41), who showed that RF clones undergo antigen-dependent affinity maturation and display somatic hypermutation profiles highly consistent with a classical germinal center origin.

T cells also play a critical role in pSS (30, 42–44). Their activation can induce inflammatory cell infiltration, B cell activation, tissue damage, and metabolic alterations. Clinically, low-dose IL-2 can exert therapeutic effects in pSS by modulating regulatory T cells (45, 46). The traditional view holds that CD4^+^ T cells are the primary pathogenic T-cell subset in pSS. However, emerging evidence suggests that CD8^+^ T cells may play an increasingly important role in its pathogenesis (47–49). Previous studies have shown that in peripheral blood, CD8^+^ T cells are elevated in patients with pSS, and the proportion of activated CD8^+^ T cells increases with higher disease activity, correlating with disease activity scores (47, 50). Upon antigen stimulation, CD8^+^ T cells become activated and differentiate into cytotoxic T lymphocytes (CTLs). These CTLs can specifically induce target cell death and tissue damage through the release of cytotoxic mediators such as perforin (PFN) and granzyme B (GzmB), or via high expression of the membrane molecule Fas ligand (FasL) (48, 51–53). MAIT cells belong to a subset of T lymphocytes characterized by a semi-invariant αβTCR, which can be activated via either the MR1-dependent pathway, which recognizes microbial riboflavin metabolites, or through cytokine-dependent signaling (54). They can produce cytokines such as IFN-γ, TNF, and IL-17, exert cytotoxic functions, and participate in antimicrobial defense and immunopathological processes (55, 56).

To further investigate changes in T cells among RF^+^ and RF^-^ pSS patients, we have analyzed the subsets of T cells. The results revealed that the proportion of CD8^+^ T cells was significantly decreased, while the proportion of MAIT cells was markedly increased in RF^-^ pSS patients, which was further confirmed by flow cytometry experiments. Functional enrichment analysis of DEGs in CD8^+^ T cells revealed strong immune activation, enhanced cytotoxicity, and hyperactive interferon signaling. This provides functional evidence that CD8^+^ T cells in RF^+^ patients are in a highly activated state, with potent cytotoxic potential and enhanced antigen recognition capacity. The upregulation of ISGs and key signaling molecules (*STAT1*, *IRF7*, *EIF2AK2*) in MAIT cells from RF^+^ patients suggests that both CD8^+^ T and MAIT subsets are in a state of strong type I interferon activation and this is further confirmed by qPCR experiments. This may be the key factor driving MAIT cell activation and altering their cytokine secretion, cytotoxic activity, and overall inflammatory profile. Concurrently, the decreased expression of the homing and retention receptors *CXCR4* and *SELL* on MAIT cells suggests that, in RF^+^ patients, these cells may more readily exit the peripheral circulation and infiltrate target organs such as the salivary glands, thereby directly participating in and exacerbating tissue immunopathology. These findings are consistent with prior research results. Wang et al. (57) investigated the quantity, immunophenotype, and function of MAIT cells in pSS patients and healthy controls. They found that MAIT cells were significantly reduced in the peripheral blood of pSS patients, and the phenotype and function of the remaining MAIT cells were altered. Furthermore, MAIT cell enrichment was increased in affected salivary gland tissues, where they influenced mucosal immunity. This suggests that the reduction of MAIT cells in peripheral blood may be due to their migration to target tissues. DAI et al. (58) further found that the expression of MAIT cells in the labial gland tissue of pSS patients was higher than HC individuals, and was even more pronounced in pSS patients experiencing symptoms such as dry mouth and rampant caries. This elevated expression may be associated with disease activity, the degree of inflammation, and antibody production. Integrating our findings, it is suggested that RF^+^ pSS patients may experience a tissue distribution remodeling of MAIT cells. The proportion and absolute number of circulating MAIT cells were significantly reduced in RF^+^ patients, which could be attributed to their activation by inflammatory mediators and subsequent infiltration into lesioned tissues to exert their effects. TCR analysis revealed differences in T-cell clonotype composition among individual samples, with a notable distinction between the RF^-^ and RF^+^ subgroups. The RF^-^ group demonstrated both generally higher TRA-chain clonal frequencies and significantly higher TCR repertoire diversity, indicating a broader and more heterogeneous T-cell response. In contrast, the significantly reduced TCR diversity in RF^+^ patients suggests that T-cell responses in this subgroup are more focused, possibly directed toward antigens associated with RF production and immune complex-mediated inflammation. This divergence in TCR diversity between RF subgroups may reflect distinct immunological contexts: RF^-^ pSS patients appear to mount a broader, polyclonal T-cell response, whereas RF^+^ patients exhibit a more constrained repertoire consistent with chronic antigen-specific stimulation.

At the level of cellular communication, the RF^+^ group demonstrated higher overall interaction strength and number, indicating that its immune microenvironment resides within a more active and complex regulatory network. Notably, Tregs exhibited enhanced signaling output to nearly all cell types, which may reflect a compensatory or pathological alteration in immune regulation. Meanwhile, MAIT cells were predicted to be important signal receivers in both RF⁻ and RF⁺ patients. Considering that MAIT cells in RF⁺ patients showed activated interferon signaling but a reduced proportion, enhanced incoming signals may contribute to their activation, functional remodeling, or redistribution toward peripheral glandular tissues. Computational ligand–receptor analysis identified the MIF signaling pathway as the dominant mediator of T–B cell collaboration in both groups, suggesting that MIF-mediated signaling may serve as a core pro-inflammatory communication axis within the pSS immune microenvironment. MIF is a pro-inflammatory cytokine and plays an important role in immune responses (59). CD74, the high-affinity receptor for MIF, is known to be involved in antigen presentation, B cell survival, and inflammatory signaling, and the MIF–CD74 axis has been implicated in multiple autoimmune conditions including rheumatoid arthritis and systemic lupus erythematosus (60). Computationally inferred pseudo-time analysis suggested that naïve B cells in RF^+^ patients were predominantly distributed in the middle-to-late stages of the reconstructed trajectory, while memory B cells exhibited a more complete inferred differentiation path. In contrast, plasma cells were enriched in the early phase of differentiation. These findings are consistent with the results from B-cell subset proportion analysis, which showed a significant increase in plasma cells in the RF^+^ group, along with upregulation of pathways related to antibody production and clonal expansion. This differentiation pattern may contribute to elevated production of autoantibodies and more severe humoral immune dysregulation. Regarding T cells, pseudo-time analysis showed that CD8^+^ T cells in the RF^+^ group were predominantly enriched in the middle-to-late stages of the trajectory, which may be associated with their more active effector state. In contrast, MAIT cells in the RF^+^ group exhibited a dispersed distribution along the trajectory, with reduced representation in both early and late phases, a pattern potentially linked to altered migratory behavior.

## 5 Conclusion

By conducting a systematic immune profiling of RF^+^ and RF^-^ pSS patients, this study reveals that RF^+^ status is closely associated with a distinct pattern of immune dysregulation. Specifically, RF^+^ patients exhibit unique immune activation features: hyperfunctional plasma cells with clonal expansion, CD8^+^ T cells with enhanced cytotoxicity and interferon responses, and MAIT cells that, despite a reduced frequency, show marked interferon signaling activation and potential tissue migration. Furthermore, the RF^+^ status is associated with a more active T-B cell collaborative network. Therefore, RF positivity may serve as a key biomarker for identifying a pSS subtype with unique immunopathological characteristics, though functional validation and longitudinal studies are needed to establish causality. This provides a new scientific perspective and potential therapeutic targets for future patient stratification, disease activity monitoring, and the development of targeted strategies for this specific subgroup.

## 6 Limitations

Several limitations of this study should be acknowledged. First, the sample size is relatively small, which may limit statistical power and generalizability. Validation in larger independent cohorts is necessary to confirm the robustness and broader applicability of our findings. Second, this study employed a cross-sectional design, capturing the transcriptomic landscape of peripheral blood mononuclear cells at a single time point. Residual confounding by unmeasured factors such as subclinical differences in disease activity cannot be entirely excluded. The differences identified between RF^+^ and RF^-^ groups represent correlative findings that require prospective validation. Third, several key analyses in this study are fundamentally computational in nature. Pseudo-time trajectory analysis reconstructs putative differentiation states based on transcriptomic similarity across simultaneously captured cells and does not constitute direct temporal observation of cellular differentiation processes; the inferred trajectories should therefore be regarded as hypothesis-generating predictions rather than confirmed differentiation pathways. Similarly, cell–cell communication results derived from CellChat are computational predictions based on known ligand–receptor interaction databases and gene expression levels, and do not represent experimentally verified signaling events. Future longitudinal cohort studies with serial sampling, combined with lineage tracing technologies, *in vitro* differentiation assays, and functional validation of predicted signaling interactions, are warranted to validate these computational inferences. Fourth, this study focused exclusively on peripheral blood mononuclear cells, and the immune landscape within target tissues such as salivary glands may differ substantially. Tissue-resident immune cell populations that do not circulate in peripheral blood could play important roles in local disease pathogenesis that are not captured by our analysis.

## Data Availability

The data reported in this paper have been deposited in the OMIX, China National Center for Bioinformation / Beijing Institute of Genomics, Chinese Academy of Sciences (https://ngdc.cncb.ac.cn/omix: accession no. OMIX016679).
